# Improving Quality of Life Through Supervised Exercise in Oncology: A Systematic Review and Meta-Analysis of Randomized Trials in Breast and Prostate Cancer

**DOI:** 10.3390/jfmk10040453

**Published:** 2025-11-20

**Authors:** Arturo Cano-Uceda, Luis De Sousa-De Sousa, Rebeca Bueno-Fermoso, Manuel Rozalén-Bustín, Carmen Lucio-Allende, Manuel Barba-Ruiz, Lara Sánchez-Barroso, José Luis Maté-Muñoz, Pablo García-Fernández

**Affiliations:** 1 Faculty of Nursing, Physiotherapy and Podiatry, Complutense University of Madrid, 28040 Madrid, Spain; 2 Faculty of Health Sciences, Alfonso X El Sabio University, 28691 Madrid, Spain; 3 Physiotherapy, Occupational Therapy and Speech Therapy Unit, Infanta Leonor University Hospital, Vallecas, 28031 Madrid, Spain; 4 InveCuid, Instituto de Investigación Sanitaria Hospital 12 de Octubre (imas12), 28041 Madrid, Spain

**Keywords:** supervised exercise, quality of life, breast cancer, prostate cancer

## Abstract

**Background:** Cancer treatments often reduce quality of life (QoL), and non-pharmacological options are limited. Supervised exercise shows promise, but its effectiveness across exercise types and patient subgroups is unclear. **Objective:** This study aimed to assess the impact of supervised exercise on QoL in breast and prostate cancer patients, considering exercise type, duration, and patient characteristics. **Methods:** A systematic review and meta-analysis including 26 randomized controlled trials (RCTs) and approximately 3500 participants was conducted according to PRISMA guidelines. PubMed, Web of Science, PEDro, SciELO, Cochrane, and Scopus were searched for randomized controlled trials (RCTs) published between 2014 and 2024. Eligible studies involved adults with breast or prostate cancer undergoing supervised exercise versus usual care or unsupervised activity. Risk of bias was assessed with the Cochrane RoB 2.0 tool, methodological quality with the PEDro scale, and certainty of evidence using the GRADE approach. **Results:** Supervised exercise was associated with significant improvements in QoL (SMD = 0.46; 95% CI: 0.22–0.70; *p* < 0.001), with considerable heterogeneity (I^2^ = 91.5%). Combined programs had the greatest effect (SMD = 0.77), followed by high-intensity interval training (HIIT) (SMD = 0.30). Shorter interventions (≤12 weeks) yielded larger improvements. Effects were more consistent in women with breast cancer. Overall, the certainty of the evidence was low. **Conclusions:** Supervised therapeutic exercise is associated with significant improvements in QoL in breast and prostate cancer patients. Combined and well-structured programs, particularly of short duration, appear especially beneficial. These findings support the integration of supervised exercise into standard oncological care. Further research should explore long-term sustainability and optimize interventions for specific patient profiles.

## 1. Introduction

Cancer remains one of the leading causes of morbidity and mortality worldwide, with nearly 20 million new cases and 9.7 million deaths estimated in 2022, according to GLOBOCAN [1]. It is expected to affect approximately one in five men and women during their lifetime and represents a major global health and economic burden [2].

Breast cancer is the most frequently diagnosed cancer in women, with an estimated 2.3 million new cases in 2022, accounting for 11.6% of all cancer diagnoses worldwide [1]. It is also one of the leading causes of cancer-related death among women, with a mortality rate of 6.9% of all cancer deaths [2,3]. At the global level, the prevalence of prostate cancer in 2021 exceeded 10 million cases, representing an increase of 189% compared to 1990, mainly due to population growth and aging [4]. Prostate cancer is the second most common cancer in men, with 1.5 million new cases and 397,000 deaths in 2022 [1,4].

The global burden is expected to continue increasing, driven by aging and improved detection [4,5]. Prostate cancer is the most common malignancy in men and the second leading cause of cancer-related death among men in the United States, with approximately 288,300 new cases and 34,700 deaths estimated for 2023 [5]. Globally, its incidence has risen markedly, with a 3.7-fold increase in new cases reported between 1990 and 2015 [6,7]. Although mortality from prostate cancer has declined in high-income countries, the disease continues to rise in both incidence and prevalence [6,7,8]. Both cancer types share modifiable risk factors such as diet, physical activity, and weight management, which may influence disease progression [9]. This underscores the importance of maintaining healthy lifestyle habits as a protective and preventive strategy against cancer [8,9].

Cancer is managed through various approaches. In breast cancer, breast-conserving surgery followed by radiotherapy is the standard treatment and is associated with a 21.7% reduction in 10-year local recurrence, a 5.4% reduction in breast cancer-specific mortality, and a 5.3% reduction in all-cause mortality at 15 years [9]. Surgical options range from conservative (lumpectomy) to radical (mastectomy), often combined with radiotherapy to lower the risk of local recurrence [10]. Systemic therapy is tailored to the tumor’s molecular subtype: hormone receptor-positive tumors are treated with endocrine therapy, HER2-positive tumors with targeted therapies such as trastuzumab, and triple-negative tumors with chemotherapy [11,12].

The management of prostate cancer is based on risk and stage stratification [13,14]. In cases of localized low-risk disease, active surveillance, comprising PSA monitoring, digital rectal examination, biopsies, and magnetic resonance imaging, is the recommended approach, with curative treatment reserved for cases showing evidence of disease progression [14]. For patients with intermediate- or high-risk disease, the main curative options are radical prostatectomy (either open or robotic) and external beam radiotherapy, both of which have been shown to improve survival [14,15]. In locally advanced or high-risk disease, the combination of radiotherapy and androgen deprivation therapy (ADT), using GnRH agonists, antagonists, or orchiectomy, is recommended, as this approach increases survival and reduces mortality [15,16,17]. In metastatic disease, ADT remains the cornerstone of treatment, often combined with next-generation androgen receptor pathway inhibitors (abiraterone, enzalutamide, darolutamide) and, in selected patients, chemotherapy (docetaxel), all of which have demonstrated survival benefits [14,15,17].

These therapies are frequently associated with adverse effects, including sarcopenia, osteoporosis, cardiotoxicity, cognitive decline, fatigue, and/or pain [18,19,20,21]. As a result, quality of life (QoL) is significantly impacted, both physically and psychologically. Chemotherapy, for instance, is linked to reduced physical function, increased fatigue, and pain, as well as other side effects that may negatively affect QoL [22]. Endocrine therapy, widely used in breast cancer, has shown persistent negative effects on QoL, particularly in postmenopausal women, impacting emotional and social well-being and contributing to pain and sleep disturbances [23]. Radiotherapy has also been associated with impaired QoL in breast cancer patients [24], while in prostate cancer, treatment-related toxicities can negatively affect urinary and gastrointestinal function [25].

Due to the substantial impact of these treatments on QoL, various non-pharmacological strategies have been investigated to mitigate adverse effects, including psychological therapies, psychosocial support, and self-management or perceived control strategies [26,27,28]. Exercise has emerged as a particularly effective intervention to enhance QoL both during and after treatment [29], improving functional capacity and reducing fatigue [30,31].

Supervised exercise programs appear to be more effective than unsupervised ones at improving health-related quality of life (HRQoL) [25,26]. Professional supervision strengthens adherence, self-efficacy, and safety through expert feedback, a consistent therapeutic alliance, and fidelity monitoring, including intensity titration, structured progression, and systematic recording of adverse events; it also ensures proper technical execution, reduces injury risk, and enables personalized prescriptions tailored to the patient’s condition and capacity [26,27,28]. As a result, the effective dose received increases and risks are reduced. By contrast, although unsupervised exercise offers greater flexibility and accessibility, it may compromise safety, adherence, and perceived exertion; with self-management predominating, dose variability increases and so does the likelihood of underdosing and dropout, potentially weakening the overall risk–benefit profile [27,28,29,30,31].

Various exercise modalities have been explored, including aerobic, resistance, and combined training, aiming to improve QoL in breast and prostate cancer patients [32,33,34,35,36,37,38,39,40]. Results have been varied, often depending on the intervention type and sometimes focusing on only one cancer type [41,42], which may introduce bias. Additionally, some studies include unsupervised exercise, home-based activity, or mind–body therapies [42,43,44] or assess unrelated outcomes such as sleep parameters [45]. Other studies included heterogeneous populations, which limits the generalizability of their findings, as they involved patients with diverse oncological disease typologies and showed substantial variability in training periodization and methodological design [29,46,47,48].

Given the substantial heterogeneity and methodological uncertainties in the existing evidence, we undertook this systematic review and meta-analysis with the end of rigorously synthesizing the current data and evaluating the impact of the supervised exercise in the quality of the patient’s life with mama and prostate cancer. Likewise, examine whether this impact varies depending on the type of exercise, the duration of the intervention, the sex and the type of cancer. This permits us to respond to the hypothesis of which the supervised therapeutic exercise produces greater improvements in the quality of life related to health than the interventions compared to adults with mama’s cancer or from the prostate within five years subsequent to the finalization of the treatment.

## 2. Materials and Methods

### 2.1. Search Strategy

This systematic review and meta-analysis was conducted in accordance with the Preferred Reporting Items for Systematic Reviews and Meta-Analyses (PRISMA) guidelines [49], following the recommendations of the Cochrane Handbook for Systematic Reviews [50], and was registered in the Open Science Framework (OSF) under the digital object identifier: https://doi.org/10.17605/OSF.IO/TCBP4.

A systematic search was carried out between September 2024 and January 2025 to identify studies evaluating the effects of exercise interventions on QoL in oncology patients. The following databases were searched: Web of Science, PubMed, PEDro, SciELO, Cochrane, and Scopus. The search was structured using the PICO framework and employed both controlled vocabulary and free-text terms. The strategy was reviewed by a health sciences librarian to ensure consistency across all databases. The search terms included: “Breast cancer,” “Prostatic cancer,” “supervised exercise,” “strength,” “aerobic,” “resistance,” “stretching,” and “Quality of life.” The specific search strategy used for PubMed is detailed in Table 1.

### 2.2. Eligibility Criteria

Two reviewers (A.C.U. and P.G.F.) independently screened the titles and abstracts of all retrieved articles to identify eligible studies. Articles whose title and abstract were relevant to the review’s objective were selected for full-text retrieval.

Studies were included if they met the following criteria: (1) Published within the last 10 years (January 2014–December 2024); (2) Written in English or Spanish; (3) Randomized controlled trials (RCTs) with a control group, in which the intervention group received supervised therapeutic exercise, including strength, cardiorespiratory, high-intensity interval training (HIIT), stretching, or a combination of these. The control group had to receive usual care or unsupervised exercise; (4) Participants were breast or prostate cancer patients who began the exercise program within five years of completing oncological treatment; (5) Cancer stages I–III with no significant comorbidities; (6) The intervention had to consist exclusively of exercise, without combination with other types of treatment (e.g., pharmacological, dietary, supplemental, psychological), aside from ongoing cancer therapy; (7) Studies had to report outcomes related to cancer-specific QoL. Exclusion criteria were as follows: (1) Animal studies; (2) Systematic reviews; (3) Non-RCTs (e.g., pilot studies, single-arm trials, non-randomized or uncontrolled trials, retrospective or cross-sectional studies) (Appendix A).

### 2.3. Data Extraction and Study Quality Assessment

Two authors (A.C.U. and P.G.F.) independently extracted data using a table adapted from the Cochrane template “Data Collection Form for Intervention Reviews: RCTs and non-RCTs”. The following information was gathered from each included study: author, year, country, cancer type, sample size, age, sex, intervention characteristics, intervention duration, measurement tool, and main outcomes. Any disagreements were resolved by a third reviewer (J.L.M.M.). Duplicate records were removed using Mendeley reference management software (version 2.100).

Risk of bias was assessed using the Cochrane Risk of Bias Tool for randomized trials (RoB 2.0) [51]. This tool evaluates bias across five domains: (1) bias arising from the randomization process; (2) bias due to deviations from intended interventions (including blinding of participants and personnel); (3) bias due to missing outcome data; (4) bias in measurement of the outcome (blinding of outcome assessors); and (5) bias in selection of the reported result. Inter-rater agreement was quantified using Cohen’s kappa (κ).

The methodological quality of the included trials was assessed using the PEDro scale [52]. This tool includes 11 criteria covering external validity (item 1, not included in the final score), internal validity (items 2–9), and statistical reporting (items 10–11). Each item is rated as “yes” or “no,” with “yes” assigned only when the criterion is clearly met. The total PEDro score is calculated from items 2 to 11, yielding a maximum score of 10. Higher scores indicate greater methodological quality. Results were categorized as follows: scores <4 = “poor,” 4–5 = “fair,” 6–8 = “good,” and 9–10 = “excellent.” Inter-rater agreement was quantified using Cohen’s kappa (κ). Both assessments (RoB 2.0 and PEDro) were conducted independently by two reviewers. Any discrepancies in either tool were resolved by consensus; if they persisted, a third reviewer adjudicated.

Certainty was assessed using the GRADE approach for each outcome and, when applicable, for subgroups. Because all included studies were RCTs, each body of evidence started at high certainty and could be downgraded across five domains: (a) Risk of bias (RoB 2.0 at the outcome level; downgraded when the weighted contribution of studies rated “high/some concerns” materially affected the estimate). (b) Inconsistency (downgraded when I^2^ was high [≥75%], point estimates were divergent and not explained by subgroups, and/or the 95% prediction interval (PI) crossed the null). (c) Indirectness (relevant differences in population, intervention, comparator, or outcome relative to the clinical question). (d) Imprecision (the 95% CI or 95% PI included the null or failed to exclude a prespecified clinically important difference for HRQoL instruments). (e) Publication bias (funnel-plot asymmetry, significant Egger’s test and/or trim-and-fill imputation that materially altered the estimate). Two reviewers independently rated certainty; discrepancies were resolved by consensus or a third reviewer.

### 2.4. Data Analysis

For each study, pre- and post-intervention data were extracted, and individual effect sizes (ES) were calculated using standardized mean differences (SMD), along with 95% confidence intervals (CIs) and standard errors (SEs), derived from means, standard deviations, and sample sizes. When needed, validated tools such as the Campbell Collaboration Effect Size Calculator and MedCalc were used to estimate SMD and SE [53,54]. When outcomes were only available as graphs, data were extracted using WebPlotDigitizer [55].

To synthesize the results quantitatively, pooled effect sizes were calculated using a random-effects model based on the Sidik–Jonkman estimator, selected for its robustness in the presence of heterogeneity [56]. The overall effect of supervised exercise versus control (usual care or unsupervised exercise) was estimated, assessing both the direction and magnitude of change. Positive SMD values indicate an improvement in QoL favoring the intervention group, while negative values indicate greater improvement in the control group. In addition to the 95% CI for the pooled effect, we report the 95% PI under a Sidik–Jonkman random-effects model with Hartung–Knapp adjustment to contextualize the expected between-study variability.

Heterogeneity across studies was evaluated using the I^2^ statistic, interpreted according to Higgins et al. [57]. 0–30% (low or insignificant), 30–50% (moderate), 50–80% (substantial), and >80% (considerable). Corresponding *p*-values and 95% CIs were also reported to better characterize the observed variability.

In studies with multiple exercise intervention arms (e.g., aerobic, resistance, or combined programs), effect sizes were calculated separately for each modality to avoid duplication of data in the pooled analysis [58]. Sensitivity analysis was performed by sequentially removing each study to identify influential or outlier studies and assess the robustness of the overall findings [59].

Subgroup analyses were conducted based on participant sex, exercise modality (aerobic, resistance, combined, or HIIT), intervention duration (≤12, 12–24, or >24 weeks), and cancer type (breast or prostate) to explore potential sources of heterogeneity and differences in effect size based on clinical and intervention-related characteristics [60]. A random-effects meta-regression was also conducted, incorporating mean age and year of publication as continuous moderator variables to assess their influence on pooled effect sizes [61]. Publication bias was evaluated using funnel plots, Egger’s regression test, and Trim-and-Fill method [62,63]. The significance level was set at *p* < 0.05. All statistical analyses were performed using SPSS software (version 30.0.0.0, IBM Corp., Armonk, NY, USA).

## 3. Results

### 3.1. Systematic Review

#### 3.1.1. Study Selection

**Figure 1:**
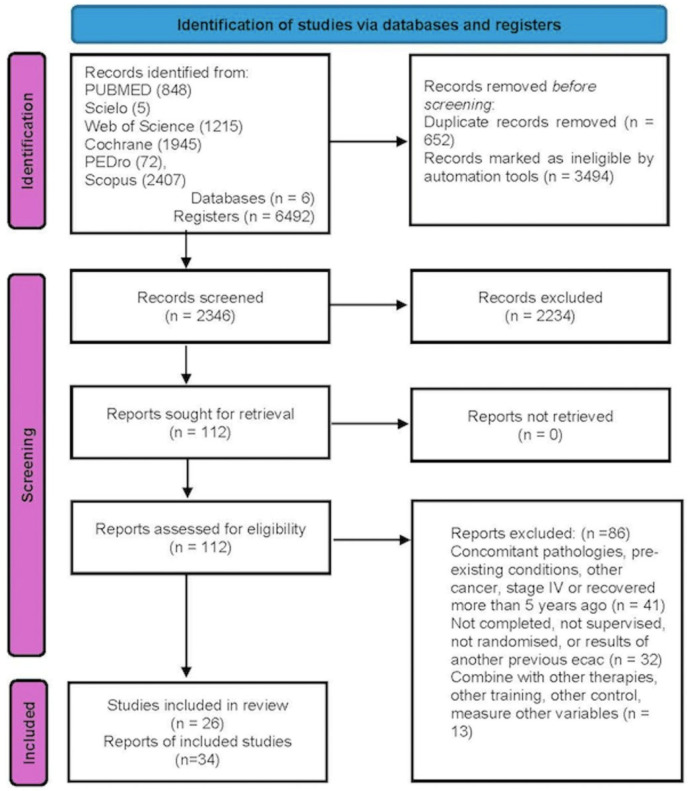
Flowchart of article selection.

The search strategy initially identified 6492 potentially eligible studies. Of these, 26 were ultimately included in the systematic review. The study selection process is illustrated in Figure 1, in accordance with the PRISMA 2020 guidelines.

#### 3.1.2. General Characteristics of Included Studies

The findings from the included studies are summarized in Appendix B, which provides detailed information on the participants, experimental conditions, and measurement tools used.

RCTs included in this review examined the effects of supervised exercise programs compared to unsupervised exercise or usual care in patients diagnosed with breast or prostate cancer. These studies were conducted across a range of countries, including the United States, Germany, Mexico, Denmark, Belgium, Brazil, Canada, and the Netherlands, providing an international perspective on the topic. A total of 26 RCTs were included, comprising a combined sample of over 3500 participants. The average age of participants in both intervention and control groups varied between studies, generally ranging from 43 to 73 years, reflecting the wide age spectrum at which these cancers typically occur.

Supervised exercise interventions included various modalities, such as aerobic training, resistance training, HIIT, and combined programs that included aerobic and resistance training. The duration of the interventions ranged widely, from 5 to 90 weeks, with training frequency most commonly set at 2 to 3 sessions per week, although some studies implemented frequencies ranging from 1 to 5 sessions per week. Although the inclusion criteria allowed for studies with interventions conducted up to five years after cancer treatment, most of the included studies implemented exercise during active oncologic treatment (Appendix C).

A variety of validated instruments were employed to assess QoL, including the FACT-B, EORTC QLQ-C30, SF-36, EORTC QLQ-PR25, FACT-P, FACT-G, EORTC QLQ-BR23, SF-12, and MQOL.

#### 3.1.3. Quality and Risk of Bias Assessment

Methodological quality was assessed using the PEDro scale. Of the 26 included PEDro scores ranged from 4 to 8, with a median of 6; 69% of studies scored ≥6 points and 27% scored ≥7, suggesting overall moderate-to-good methodological quality. As is typical in exercise interventions, blinding items contributed fewer points. Inter-rater agreement was almost perfect (Cohen’s kappa = 0.86) (Table 2).

**Figure 2:**
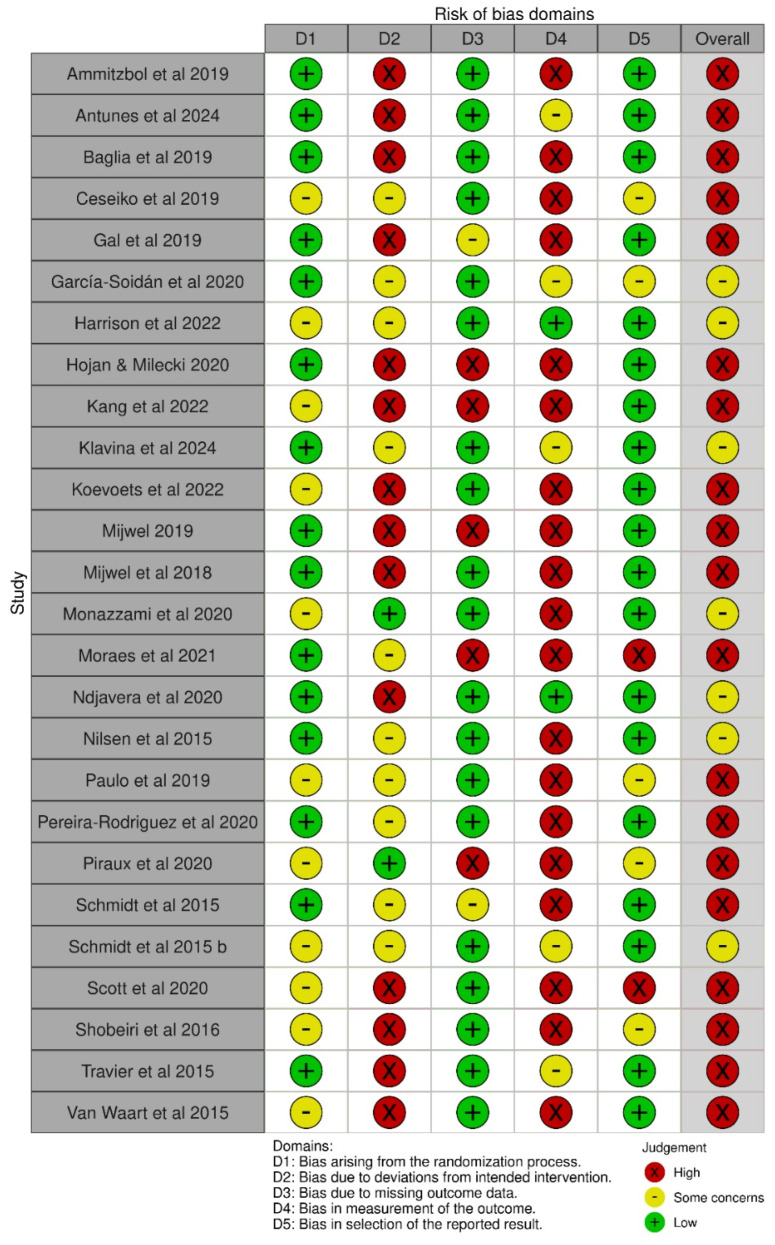
Risk of bias assessment: summary for individual studies [30,33,34,35,36,37,38,39,41,64,65,66,67,68,69,70,71,72,73,74,75,76,77,78,79,80].

**Figure 3:**
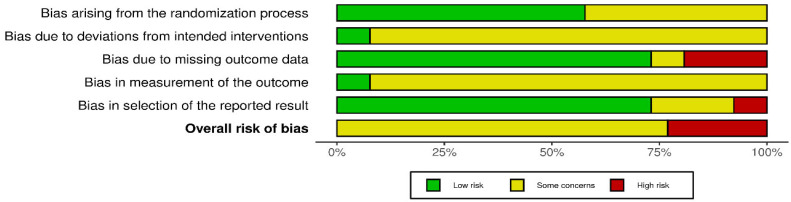
Risk of bias assessment: aggregate appraisal results.

Regarding the risk of bias, evaluated using the Cochrane RoB 2.0 tool, 7 studies (25.9%) were judged to have a high risk of bias, 19 studies (70.4%) had some concerns, and only 1 study (3.7%) assessed as having a low risk of bias. Inter-rater agreement was substantial (Cohen’s kappa = 0.74) (Figure 2 and Figure 3).

The certainty of the evidence regarding the effects of supervised therapeutic exercise on quality of life in cancer patients was assessed using the GRADE methodology (Table 3). Overall, supervised exercise interventions showed a small-to-moderate beneficial effect compared to the control group (SMD = 0.46; 95% CI: 0.22 to 0.70), although the certainty of the evidence was rated as low, partly due to risk of bias. When analyzed by type of exercise, high-intensity interval training (HIIT) was associated with moderate certainty of evidence, with consistent findings and no serious imprecision (SMD = 0.30; 95% CI: 0.10 to 0.49). In contrast, combined exercise interventions, although showing a potentially greater effect (SMD = 0.77; 95% CI: 0.20 to 1.34), were rated as very low certainty due to serious concerns in multiple domains, including risk of bias, inconsistency across studies, and imprecision. Aerobic and resistance training modalities also yielded very low certainty, primarily due to small sample sizes and wide confidence intervals, limiting the precision and generalizability of the results. This bias is influenced by the inherent nature of exercise interventions, which precludes blinding of participants and personnel, potentially affecting the perception and reporting of subjective outcomes such as quality of life.

### 3.2. Data Synthesis

#### 3.2.1. Meta-Analysis

**Figure 4:**
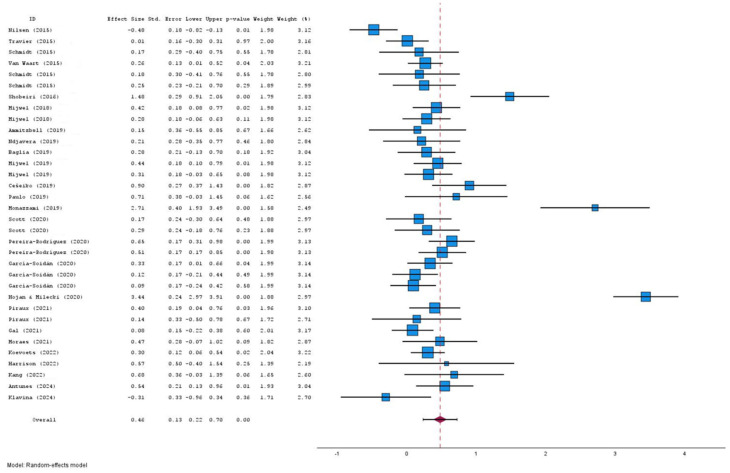
Forest Plot [30,33,34,35,36,37,38,39,41,64,65,66,67,68,69,70,71,72,73,74,75,76,77,78,79,80].

The intervention showed a positive effect of moderate magnitude, although individual study results were highly variable. The pooled effect size (ES) for supervised exercise versus the control group on cancer patients’ QoL was 0.46 (95% CI: 0.22–0.70; I^2^ = 91.5%; 95% PI: −0.92–1.87; τ^2^ s≈ 0.45; *p* < 0.001) (Figure 4).

#### 3.2.2. Sensitivity and Subgroup Analysis

Sensitivity analysis, performed sequentially excluding each study individually, confirmed the robustness of the results. The estimated effect sizes did not change substantially, ranging from 0.359 to 0.492, and remained statistically significant in all cases. No single study notably altered the overall effect in terms of direction or magnitude. Heterogeneity remained high (I^2^ = 89–92%), indicating persistent variability among the included studies (Table 4).

Subgroup differences are presented descriptively, without statistical testing, due to insufficient power and the risk of multiplicity. Exercise interventions that combined aerobic and resistance training showed the greatest effect on QoL, although they were associated with very high heterogeneity, indicating considerable variability among studies. HIIT programs also demonstrated a significant effect, with moderate heterogeneity, suggesting greater consistency across results. In contrast, endurance-based programs yielded non-significant results and were associated with high heterogeneity. Resistance training programs showed a small, non-significant effect, accompanied by substantial heterogeneity (Table 5).

A subgroup analysis based on program duration revealed that shorter interventions (up to 12 weeks) produced the largest effect, although they were associated with high heterogeneity. Programs lasting 12 to 24 weeks and those exceeding 24 weeks showed smaller effects, but with lower variability across studies (Table 5).

Subgroup analysis by participant sex showed a significant effect in women, although heterogeneity remained high. In studies conducted with male participants, the effect size was larger but not statistically significant, and heterogeneity was extremely high, limiting the interpretability of results within this subgroup. Since breast cancer studies were conducted exclusively in women and prostate cancer studies exclusively in men, the results of the cancer type subgroup analysis mirror those of the sex-based analysis (Table 5).

The meta-regression analysis found no statistically significant associations between effect size and either participant age or year of publication (Table 6).

#### 3.2.3. Publication Bias

**Figure 5:**
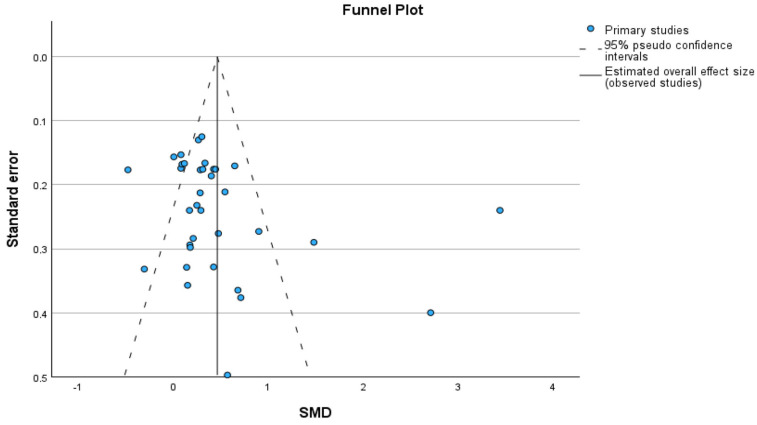
Funnel plot assessing publication bias. Egger’s test and Duval and Tweedie’s trim-and-fill.

Publication bias was assessed using a funnel plot, Egger’s test, and Duval and Tweedie’s trim-and-fill method. The funnel plot showed an approximately symmetrical distribution of studies around the pooled effect (Figure 5). Egger’s test did not indicate small-study effects (intercept = −0.092; 95% CI −0.827 to 0.643; two-sided *p* = 0.801; k = 34). Trim-and-fill did not impute any studies, and the pooled SMD remained unchanged (0.463; 95% CI 0.222–0.703), which is consistent with the absence of publication-related asymmetry (Table 7).

## 4. Discussion

This systematic review and meta-analysis aimed to evaluate the effectiveness of supervised therapeutic exercise in improving QoL in patients with breast or prostate cancer. A total of 34 interventions from 26 RCTs were included, of which 32 reported significant improvements in QoL following the intervention. The review period and the inclusion of patients within five years after completing primary treatment were established to reflect the contemporary era of oncological care and exercise interventions, focusing on early survivorship when treatment effects remain modifiable, thereby minimizing methodological and historical heterogeneity.

The observed ES was 0.46, corresponding to a small-to-moderate effect according to conventional thresholds, which confirms our hypothesis that supervised therapeutic exercise improves QoL compared with unsupervised interventions or usual care. This finding is clinically relevant given the commonly impaired quality of life in this population and the limited availability of effective pharmacological treatments to address it; however, the high heterogeneity and low certainty of the evidence suggest that these results should be interpreted with caution. These findings are consistent with previous studies [30,70,72] and provide strong support for the use of therapeutic exercise as a safe and effective intervention. The effectiveness of exercise may partly be attributed to its capacity to mitigate various side effects of oncological treatment, such as cardiotoxicity, peripheral neuropathy, dyspnea, and cognitive impairment, through mechanisms like improved cardiovascular function and modulation of systemic inflammation [65]. In addition, exercise induces meaningful physiological changes, including improved body composition, increased muscular strength, and enhanced aerobic capacity, all of which are directly associated with greater physical functionality and more favorable health perceptions [27]. To clinically contextualize our ES, back-translation into original units suggests that, on 0–100 scales such as the EORTC QLQ-C30 and SF-36, it corresponds to approximately 7–9 points, and on the FACT-G to around 5–7 points. These changes are at or above commonly reported MCIDs. Thus, beyond statistical significance, the average effect is likely clinically meaningful at the group level; nevertheless, its interpretation should consider the specific domain assessed, the population’s standard deviation and the clinical context, as well as the heterogeneity observed across studies.

Supervised exercise also provides psychological benefits, consistently reducing anxiety and depression while enhancing self-esteem and emotional well-being [26]. These effects are amplified in group-based formats, which promote social interaction and emotional support, further contributing to a more positive experience during disease process [26]. Moreover, professional supervision emerges as a key factor in maximizing the benefits of exercise. Supervised interventions have been shown to be more effective than unsupervised ones [67,71], because they allow for proper individualization based on the patient’s needs and capacities [26], enhance adherence and motivation [27], and ensure correct technical execution, thereby optimizing outcomes and minimizing injury risk [26]. Our findings are consistent with current exercise prescription guidelines in oncology (e.g., ACSM), which recommend 150 min/week of moderate-intensity aerobic activity (or 75 min vigorous) and 2–3 days/week of resistance training, with appropriate progression and safety screening [81]. Supervised delivery supports individualized programming, fidelity monitoring, and adverse-event surveillance, and is often associated with high adherence (typically 80–90%, frequently ≥85%), reinforcing its practical value for implementation. However, although directional consistency supports the utility of supervised exercise, the narrow publication window and the high heterogeneity restrict generalizability. Applicability appears greater in high-income settings, where structured supervision is feasible; extrapolation to resource-limited contexts may be limited.

Two studies reported negative effects on QoL, although they did not significantly alter the overall pooled effect: Klavina et al. [71] and Nilsen et al. [39]. These outcomes may be attributed to specific study designs or clinical contexts that negatively impacted the exercise–QoL relationship. In Klavina et al., despite improvements in physical symptoms and fatigue, global QoL declined, possibly due to the unsupervised format, remote follow-up, higher incidence of peripheral neuropathy, and lack of complementary support, all of which may have exacerbated emotional distress during intensive treatment. In the study by Nilsen et al. [39], no improvements in QoL were observed despite gains in physical function. This may be attributed to high baseline QoL, the brief duration of the intervention, and a common dissociation between physical improvement and subjective well-being. These findings underscore the importance of aligning exercise programs not only with physiological goals but also with the clinical context, supervision level, and characteristics of the target population to achieve meaningful impacts on QoL.

Conversely, some studies reported atypically high effect sizes, likely influenced by methodological or contextual factors. In the study by Monazzami et al. [74], sample homogeneity in postmenopausal women with stage I breast cancer, may have led to more uniform responses, and the exclusive use of the McGill questionnaire, possibly more sensitive to change, may have amplified the detected effects. In the study by Shobeiri et al. [41], low baseline QoL provided greater room for improvement, and the fact of being a supervised group-based intervention, applied post-treatment, may have enhanced both physical and psychosocial recovery. Additionally, control group inactivity, resource-limited settings, and expectation bias may have contributed to the results. Similarly, in the study by Hojan and Milecki [69], the intervention was implemented during a clinically critical period (radiotherapy combined with androgen deprivation therapy in men with high-risk prostate cancer), which helped mitigate a functional and emotional decline that was observed in the control group. The acute phase follow-up and small sample size may have amplified these effects in all three cases.

An important limitation of exercise interventions lies in the impossibility of blinding participants and personnel involved in supervised therapeutic exercise programs. This situation, inherent to behavioral interventions, may influence the perception and subjective reporting of outcomes, particularly when assessing dimensions such as quality of life. This risk of detection and performance bias is widely recognized in methodological literature and helps explain why the certainty of the evidence according to GRADE is low or very low in several subgroups and moderate where greater consistency and precision are present. Clinically, this means that additional research is likely to modify the estimate effect; indeed, a low GRADE rating indicates that future evidence may change our conclusions and that recommendations should be cautious. With very low GRADE, uncertainty is substantial and the true effect could differ materially; in this scenario, recommendations should be conditional, emphasizing shared decision-making, the patient’s context and preferences, and prioritizing the individualization of dose and supervision while higher-quality data are generated. Although strategies such as blinding outcome assessors or using objective measures can mitigate these biases, they are not always applicable or sufficient when dealing with self-reported outcomes.

Sensitivity analysis demonstrated high stability of the overall effect, with very similar effect sizes after exclusion of each study, and confidence intervals that consistently excluded the null value. Heterogeneity remained high and stable, except in the case of Hojan & Milecki [69], whose exclusion reduced I^2^ to 82.1%, suggesting a possible isolated contribution to variability. Overall, these findings reinforce the robustness of the meta-analysis and rule out any disproportionate influence of individual studies.

Heterogeneity was very high, with an I^2^ of 91.5%, an τ^2^ of 0.47 based on 34 estimates, and a PI of −0.92 to 1.87 in SMD, indicating that in future studies the effect may range from null or slightly unfavorable to clearly favorable. This variability consists of clinical differences among patients; variation in exercise modality, dose, intensity, and progression; differing levels of supervision and adherence; heterogeneous comparators; and diversity in instruments and timing of health-related quality-of-life assessment, as well as methodological limitations such as lack of blinding and loss to follow-up. No single factor explains the observed variation, so subgroup results should be interpreted as exploratory, and it is recommended to individualize dose and supervision and to promote multicenter trials with standardized dose and fidelity.

Subgroup analysis by exercise type suggested clinically relevant differences in effectiveness. Combined exercise interventions showed the strongest effects, although with high variability between studies, which may be attributed to differences in implementation or participant characteristics. Compared to aerobic- or resistance-only programs, combined training has been linked to greater improvements in QoL, particularly in physical function, general health, mental health, and vitality [74]. According to Xiong et al. [82], this approach may be the most effective for improving health-related QoL in cancer survivors, likely due to the synergistic effects of aerobic and resistance training.

HIIT also showed consistent and homogeneous effects, with moderate improvements in QoL. Its effectiveness may be attributed to substantial improvements in cardiorespiratory fitness, which is closely associated with health-related QoL [83], as well as reductions in cancer-related fatigue and improvements in other relevant domains [84]. Furthermore, low-volume HIIT has proven to be feasible, safe, and well tolerated, even in patients with advanced-stage cancer, with no serious adverse events reported [85]. In contrast, interventions based solely on aerobics or resistance training yielded more modest and inconclusive results. These findings highlight the importance of considering not only the inclusion of exercise but also its modality, intensity, and structure when planning effective interventions for this population.

Subgroup analysis by program duration suggests that intervention length influences both the magnitude and consistency of effects. Shorter programs (≤12 weeks) tended to produce greater improvements, while with greater variability, potentially due to differences in intensity, frequency, or adherence. In contrast, longer interventions demonstrated more modest but consistent effects, possibly reflecting a plateau in benefits or a decline in sustained intensity over time. One possible explanation is that shorter programs foster higher motivation and adherence [86], increasing patient engagement and enhancing both objective outcomes and perceived benefits. For instance, supervised therapeutic exercise programs combining strength, aerobic, and stretching components have shown significant improvements in QoL within just six weeks [40]. Moreover, even brief interventions can induce meaningful physiological and psycho-emotional adaptations [87,88], with a positive impact on QoL. Programs lasting 12 to 24 weeks showed similar levels of heterogeneity but smaller effect sizes, which may be due to accumulated physical and emotional fatigue affecting perception of benefits. These findings emphasize the importance of balancing duration, intensity, and adherence, and suggest that short, well-structured, and intensive interventions can be particularly effective when coupled with proper follow-up. The greater magnitude observed in shorter and/or combined programs is clinically plausible (better adherence, higher effective dose, and fidelity), but it should be interpreted as an exploratory trend since no interaction tests were performed. The apparent inverse relationship between duration and effect generates hypotheses to be tested in future randomized controlled trials with standardized dose/intensity and follow-up.

Subgroup analysis by sex revealed differences in effect size and consistency, with more robust and homogeneous results in women. However, since all studies involving women included breast cancer patients, and those involving men focused exclusively on prostate cancer, these differences may reflect both sex and cancer type. Exercise benefits appear more consistent in women with breast cancer, whereas results in men with prostate cancer were more variable and less conclusive. One possible explanation is that side effects of breast cancer treatment, such as fatigue and depression, are more amenable to exercise-based interventions. In contrast, androgen deprivation therapy used in prostate cancer is associated with more severe side effects, such as muscle loss and sexual dysfunction [89,90,91]. Additionally, women may benefit more from the psychosocial dimensions of exercise, such as social support or improved body image, which positively impact QoL. These psychosocial benefits appear less pronounced in men. The close link between sex and tumor type complicates interpretation, and future studies are needed to disentangle the roles of biological, treatment-related, and tumor-specific factors.

The meta-regression analysis did not find any significant associations between exercise effect size and either the mean age of participants or the year of study publication. These results suggest that neither participant age nor potential time-related changes in study design or quality have systematically influenced the observed outcomes.

Finally, assessment of publication bias did not reveal any significant concerns that could compromise the reliability of the findings. The funnel plot showed relatively symmetrical dispersion of studies around the pooled estimate, with no clear accumulation of small studies reporting positive results, suggesting no major asymmetrical. This visual impression was supported by a non-significant Egger’s test, indicating no statistical evidence of publication bias. Similarly, the Trim and Fill method did not impute any missing studies, reinforcing the robustness of the findings and suggesting that the observed effect is unlikely to be skewed by systematic non-reporting of negative or unpublished studies. Given the high level of between-study heterogeneity, these analyses should be interpreted with appropriate caution. In addition, in the subgroup analyses, the number of studies per stratum was small, which limits the power of Egger’s test and the trim-and-fill method; consequently, subgroup findings are considered exploratory and should be interpreted with caution.

The results of this review provide preliminary support for considering the incorporation of structured, supervised exercise programs as a possible complementary therapy in the care of patients with breast and prostate cancer. The findings of this systematic review and meta-analysis appear to confirm the effectiveness of supervised therapeutic exercise in improving quality of life among patients with breast and prostate cancer. Combined aerobic and resistance training programs, particularly those of shorter duration, showed the greatest benefits. Supervised exercise should be integrated into oncology care and prescribed by qualified professionals within multidisciplinary teams to ensure safety and individual adaptation. Incorporating supervised exercise into clinical pathways may optimize physical function, psychological well-being, and treatment tolerance. In addition, implementing hospital- and community-based exercise programs could improve accessibility, adherence, and continuity of physical activity after cancer treatment. Future research should aim to reduce the observed heterogeneity by standardizing exercise protocols in terms of duration, intensity, frequency, and level of supervision. High-quality randomized controlled trials with extended follow-up periods are needed to assess the long-term sustainability of benefits and to determine the most effective modalities according to cancer type, stage, and patient characteristics. Further studies in prostate cancer populations are particularly warranted, as they remain underrepresented in the literature. Future investigations should also incorporate objective measures of physical function, biomarkers, and cost-effectiveness analyses to reinforce the clinical applicability of supervised exercise. Finally, mechanistic and implementation studies are essential to elucidate the biological and contextual factors underlying the effectiveness of exercise interventions in oncology practice.

This meta-analysis has several limitations that should be considered when interpreting the results. First, restricting the search to 2014–2024 may introduce temporal bias by omitting earlier RCTs; this decision was adopted as a trade-off to enhance applicability to current practice and reduce historical heterogeneity. Our ≤5-year threshold does not imply a stable recovery phase; it encompasses residual effects and early recovery. Although it improves internal validity and clinical comparability, it may limit generalizability to long-term survivors. The RCT-only criterion increases internal validity at the cost of potentially reduced external validity. The substantial heterogeneity among studies, stemming from differences in study populations, the type and duration of exercise, and assessment tools, may have influenced the effect estimates. Although subgroup analyses were performed, the limited number of studies within certain categories reduced their explanatory power. Moreover, the lack of blinding and incomplete reporting of randomization procedures in several trials introduce a potential risk of bias. The absence of long-term follow-up data precludes assessment of adherence and the residual effects of exercise. Finally, it is important to note that most included studies were conducted in high-income countries, which may restrict the applicability of the results to settings with different healthcare infrastructures, more limited access to supervised programs, or distinct sociocultural contexts.

## 5. Conclusions

Overall, supervised exercise is associated with small-to-moderate improvements in health-related quality of life among cancer survivors. However, these estimates should be interpreted with caution due to between-study heterogeneity and because, according to GRADE, the certainty of the evidence is low or very low in most comparisons. The applicability of the findings is greater in high-income settings, where structured supervision is feasible; generalizability to resource-limited contexts may be reduced.

On this basis, we should consider integrating supervised exercise where local resources allow it, prioritizing individualization, safety monitoring, and adherence, and embedding it within shared decision-making. High-quality, multicenter randomized controlled trials with standardized dose and adherence, long-term follow-up, and implementation and cost-effectiveness evaluation are required to confirm sustainability and to determine who benefits most and under what conditions.

## Figures and Tables

**Table 1:** Search strategy—PubMed.

Pub Med—Search Strategy:	Natural Terms and Equations	Results Obtained
1	(“Breast Neoplasms” [MeSH Terms] OR “breast cancer” [Title/Abstract]) OR (“Prostatic Neoplasms” [MeSH Terms] OR “prostate cancer” [Title/Abstract])	
2	(“Exercise” [MeSH Terms] OR “Exercise Therapy ” [MeSH Terms] OR “supervised exercise” [Title/Abstract] OR “aerobic” [Title/Abstract] OR “resistance” [Title/Abstract] OR “strength” [Title/Abstract] OR “HIIT” [Title/Abstract] OR “stretching” [Title/Abstract])	
3	(“Quality of Life” [MeSH Terms] OR “quality of life” [Title/Abstract] OR “QoL” [Title/Abstract])	
4	1 AND 2 AND 3	
		848

**Table 2:** Methodological quality measured by PEDro scale.

Study	1	2	3	4	5	6	7	8	9	10	11	Total
Ammitzbøll et al. 2019 [38]	+	+	−	+	−	−	−	−	+	+	+	5
Antunes et al. 2024 [30]	+	+	+	+	−	−	−	+	+	+	+	7
Baglia et al. 2019 [64]	+	+	+	+	−	−	−	+	+	+	+	7
Cešeiko et al. 2019 [65]	+	+	−	+	−	−	−	+	−	+	+	5
Gal et al. 2021 [66]	+	+	−	+	−	−	−	−	+	+	+	5
García-Soidán et al. 2015 [67]	+	+	+	+	−	−	+	+	−	+	+	7
Harrison et al. 2022 [68]	−	+	−	+	−	−	+	+	−	+	+	6
Hojan & Milecki 2020 [69]	+	+	+	+	−	−	−	+	+	+	+	7
Kang et al. 2022 [70]	+	+	−	+	−	−	−	+	+	+	+	6
Klavina et al. 2024 [71]	+	+	+	+	−	−	−	−	−	+	+	5
Koevoets et al. 2022 [72]	−	+	−	+	−	−	−	+	+	+	+	6
Mijwel et al. 2018 [73]	+	+	−	+	−	−	−	−	−	+	+	4
Mijwel et al. 2019 [36]	+	+	−	+	−	−	−	+	+	+	+	6
Monazzami et al. 2020 [74]	+	+	−	+	−	−	−	−	−	+	+	4
Moraes et al. 2021 [75]	+	+	−	+	−	−	−	+	+	+	+	6
Ndjavera et al. 2020 [37]	+	+	+	+	−	−	+	−	+	+	+	7
Nilsen et al. 2015 [39]	+	+	+	+	−	−	+	−	+	+	+	7
Paulo et al. 2019 [76]	−	+	−	+	−	−	−	−	+	+	+	5
Pereira-Rodríguez et al. 2020 [77]	+	+	+	+	−	−	+	−	−	+	+	6
Piraux et al. 2020 [78]	−	+	−	+	−	−	−	+	+	+	+	6
Schmidt et al. 2015 [33]	+	+	+	−	−	−	−	−	−	+	+	5
Schmidt et al. 2015 [79]	+	+	−	+	−	−	−	+	+	+	+	6
Scott et al. 2020 [80]	−	+	−	+	−	−	−	+	+	+	+	6
Shobeiri et al. 2016 [41]	+	+	−	+	−	−	−	+	+	+	+	6
Travier et al. 2015 [34]	+	+	+	+	−	−	+	+	+	+	+	8
Van Waart et al. 2015 [35]	+	+	−	+	−	−	−	+	+	+	+	6

+ = criterion met; − = criterion not met. 1. Eligibility criteria and source (this item does not contribute to the total score).; 2. Random allocation; 3. Concealed allocation; 4. Baseline comparability; 5. Blinding of participants; 6. Blinding of therapists; 7. Blinding of assessors; 8. Adequate follow-up (>85%); 9. Intention-to-treat analysis; 10. Between-group statistical comparisons; 11. Reporting of point measures and measures of variability.

**Table 3:** GRADE Assessment of Supervised Exercise Overall and by Type vs. Control for Quality of Life in Cancer Patients.

Certainty Assessment							№ of Patients		Effect	Certainty	Importance
№ of Studies	Study Design	Risk of Bias	Inconsistency	Indirectness	Imprecision	Other	Therapeutic Exercise	Control	SMD (95% CI)		
**Supervised Exercise vs. Control for Quality of Life** (Include all supervised exercise types)											
26 *	Randomized trials	serious	serious	not serious	not serious	none	1781/3502 (51%)	1721/3502 (49%)	**SMD 0.46** (0.22 to 0.70)	⨁⨁◯◯ Low	Important
**Supervised HIIT vs. Control for Quality of Life**											
**8**	Randomized trials	serious	not serious	not serious	not serious	none	424/800 (53.0%)	376/800 (47.0%)	**SMD 0.30** (0.10 to 0.49)	⨁⨁⨁◯ Moderate	Important
**Supervised Combined Exercise vs. Control for Quality of Life**											
**14**	Randomized trials	serious	serious	not serious	serious	none	789/1559 (50.6%)	770/1559 (49.4%)	**SMD 0.77** (0.20 to 1.34)	⨁◯◯◯ Very low	important
**Supervised Aerobic Exercise vs. Control for Quality of Life**											
**4**	Randomized trials	serious	serious	not serious	serious	none	243/488 (49.8%)	245/488 (50.2%)	**SMD 0.42** (−0.08 to 0.92)	⨁◯◯◯ Very low	Important
**Supervised Resistance exercise vs. Control for Quality of Life**											
**8**	Randomized trials	serious	serious	not serious	serious	none	325/655 (49.6%)	330/655 (50.4%)	**SMD 0.20** (−0.05 to 0.46)	⨁◯◯◯ Very low	Important

* Include 34 interventions. CI: confidence interval. SMD: standardized mean difference. ⨁⨁⨁⨁ = High certainty; ⨁⨁⨁◯ = Moderate certainty; ⨁⨁◯◯ = Low certainty; ⨁◯◯◯ = Very low certainty.

**Table 4:** Sensitivity Analyses.

Author. Year	Exercise	ES	LL	UL	I2
**Nilsen et al. (2015) [39]**	R	0.492	0.251	0.733	91.2
**Travier et al. (2015) [34]**	C	0.478	0.231	0.724	91.5
**Schmidt et al. (2015) [33]**	R	0.470	0.220	0.717	91.8
**Van Waart et al. (2015) [35]**	C	0.470	0.222	0.717	91.4
**Schmidt et al. (2015) [79]**	R	0.471	0.224	0.718	91.8
**Schmidt et al. (2015) [79]**	A	0.471	0.222	0.720	91.4
**Shobeiri et al. (2016) [41]**	A	0.434	0.193	0.674	91.3
**Mijwel et al. (2018) [73]**	R-HIIT	0.465	0.217	0.712	91.7
**Mijwel et al. (2018) [73]**	A-HIIT	0.463	0.222	0.703	91.5
**Ammitzbøll et al. (2019) [38]**	R	0.471	0.225	0.718	91.8
**Ndjavera et al. (2019) [37]**	C	0.470	0.223	0.718	91.8
**Baglia et al. (2019) [64]**	C	0.469	0.221	0.716	91.7
**Mijwel et al. (2019) [36]**	A-HIIT	0.468	0.220	0.716	91.6
**Mijwel et al. (2019) [36]**	R-HIIT	0.464	0.216	0.712	91.7
**Cešeiko et al. (2019) [65]**	R	0.451	0.204	0.697	91.7
**Paulo et al. (2019) [76]**	C	0.457	0.210	0.704	91.9
**Monazzami et al. (2019) [74]**	C	0.405	0.191	0.619	89.1
**Scott et al. (2020) [80]**	AI	0.468	0.221	0.716	91.8
**Scott et al. (2020) [80]**	AC	0.472	0.225	0.719	91.8
**Pereira-Rodríguez et al. (2020) [77]**	C	0.458	0.210	0.705	91.6
**García-Soidán et al. 2015 (2020) [67]**	R	0.467	0.220	0.715	91.6
**Pereira-Rodríguez et al. (2020) [77]**	HIIT	0.475	0.228	0.722	91.6
**García-Soidán et al. 2015 (2020) [67]**	A	0.475	0.228	0.722	91.5
**García-Soidán et al. 2015 (2020) [67]**	C	0.474	0.227	0.721	91.6
**Hojan & Milecki** **(2020) [69]**	C	0.359	0.189	0.529	82.1
**Piraux et al. (2021) [78]**	HIIT	0.465	0.217	0.713	91.7
**Piraux et al. (2021) [78]**	R	0.472	0.225	0.719	91.8
**Gal et al. (2021) [66]**	C	0.475	0.229	0.722	91.5
**Moraes et al. (2021) [75]**	R	0.463	0.215	0.711	91.8
**Koevoets et al. (2022) [72]**	C	0.469	0.221	0.716	91.3
**Harrison et al. (2022) [68]**	C	0.461	0.214	0.708	91.9
**Kang et al. (2022) [70]**	HIIT	0.458	0.211	0.705	91.9
**Antunes et al. (2024) [30]**	C	0.461	0.213	0.709	91.7
**Klavina et al. (2024) [71]**	HIIT	0.484	0.240	0.727	91.6

ES: Effect size; LL: Lower limit; UL: Upper limit: A: Aerobic; C: Combined; R: Resistance; HIIT: High-Intensity Interval Training; AC: Continuous Aerobic; AI: Incremental Aerobic.

**Table 5:** Subgroup analysis by Type of Exercise, Duration of the Exercise Program and Gender.

Exercise	ES	LL	UL	I^2^
**Endurance**	0.424	−0.079	0.926	82.5
**Combined**	0.771	0.204	1.338	96.2
**HIIT**	0.301	0.103	0.499	49.4
**Resistance**	0.201	−0.054	0.457	64.5
**Duration**	**ES**	**LL**	**UL**	**I^2^**
**≤12 weeks**	0.846	0.266	1.426	94.1
**12–24 weeks**	0.235	0.069	0.400	54.0
**>24 weeks**	0.253	0.059	0.447	59.3
**Gender**	**ES**	**LL**	**UL**	**I^2^**
**Men**	0.711	−0.234	0.882	95.8
**Women**	0.391	0.198	0.583	84.4

ES: Effect size; LL: Lower limit; UL: Upper limit; HIIT: High-Intensity Interval Training.

**Table 6:** Meta-regression analysis.

	Coefficient	*p*-Value
**Age**	0.005	0.742
**Year of Publication**	0.021	0.687

**Table 7:** Assessment of Publication Bias.

	Main Result	*p*-Value	95% CI
**Egger’s test**	−0.092	0.801	−0.827 to 0.643
**Trim and Fill**	Imputed studies = 0 Effect size = 0.463	<0.001	0.222 to 0.703

IG: intervention group. CG: control group. EORTC QLQ-C30 = European Organisation for Research and Treatment of Cancer Quality-of-Life Questionary Core 30. SF-36 = Short Form 36. FACT-B = Functional Assessment of Cancer Therapy—Breast. FACT-P = Functional Assessment of Cancer Therapy—Prostate. FACT-G = Functional Assessment of Cancer Therapy—General. EORTC QLQ-BR23 = European Organisation for Research and Treatment of Cancer Quality of Life Questionnaire Breast Cancer. SF-12 = Short Form-12. MQOL = McGill Quality of Life Questionnaire. RT = resistance training. AT = aerobic training. HIIT = High-intensity Interval training. MICT = Moderate-Intensity Continuous Training. ET = Endurance Training. LET = Linear Exercise Therapy. NLET = Non-Linear Exer.

Note: The “✔” symbol indicates compliance with the corresponding feature.

## Data Availability

No new data were created or analyzed in this study. Data sharing is not applicable to this article.

## Appendix A. PICOS Framework Used to Define the Inclusion and Exclusion Criteria of the Review

**PICOS** **Category**	**Inclusion Criteria**	**Exclusion Criteria**
P (Population)	Patients diagnosed with stage I-III breast or prostate cancer who began the training program no later than 5 years after completing treatment.	Patients with stage IV or metastatic disease, with concomitant pathologies or pre-existing conditions, or who have another type of cancer or completed treatment more than 5 years ago.
I (Intervention)	Supervised therapeutic exercise program that included modalities such as strength training, cardiorespiratory training, HIIT, stretching, or a combination of these.	Usual care, unsupervised training, or supervised training combined with some other type of intervention not specifically targeted at cancer.
C (Comparators)	Regular care or unsupervised training	Control groups that received other types of training
O (Outcomes)	Studies reporting cancer-specific quality of life (QoL) outcomes assessed using validated instruments (e.g., EORTC QLQ-C30, FACT-G, or similar).	Studies measuring other primary variables other than QoL, incomplete results
S (Study Design)	Randomized Controlled Trials (RCTs) with a control group. Published between January 2014 and December 2024 (last 10 years).	Systematic reviews. Studies that are not RCTs (e.g., pilot studies, single-arm trials, non-randomized or non-controlled trials, retrospective or cross-sectional studies)

## Appendix B. The Included Studies

**Author/** **Year**	**Country**	**Cancer Type**	**Sample Size**	**Age (SD)**	**Sex**	**Intervention Characteristics**	**Duration of Intervention**	**Measure Tool**	**Results**
Schmidt et al. 2015 [33]	Germany	Breast	IG: 49 CG: 46	IG: 52.2 (9.9) CG: 53.3 (10.2)	W	IG: RT CG: active structured controls (relaxation)	12 weeks 2 days/week	EORTC QLQ-C30	IG Pre: 61.5 ± 17.5 Post: 61.7 ± 18.3 CG Pre: 59.4 ± 16.6 Post: 54.9 ± 22.9
Schmidt et al. 2015 [79]	Germany	Breast	GRT: 21 GET: 20 CG: 26	RT: 53 (12.55) ET: 56 (10.15) CG: 54 (11.19)	W	RT: RT ET: AT CG: Passive Usual Care (no exercise/maintain habitual activity/no specific advice)	12 weeks 2 days/week	EORTC QLQ-C30	GRT Pre: 24.8 ± 14.0 Post: 31.3 ± 18.0 GET Pre: 30.4 ± 18.2 Post: 36.7 ± 17.8 CG Pre: 31.1 ± 19.7 Post: 31.0 ± 15.3
Travier et al. 2015 [34]	Netherlands	Breast	IG: 102 CG: 102	IG: 49.7 (8.2) CG: 49.5 (7.9)	W	IG: Combined CG: Passive Usual Care (no exercise/maintain habitual activity/no specific advice)	18 weeks 2 days/week	EORTC QLQ-C30 SF-36	IG Pre: 74.8 ± 20.4 Δ change IC95% −4.5 [−8.4 to −0.3] CG Pre: 72.5 ± 19.4 Δ change IC95% −4. [−8.8 to −0.3]
Van Waart et al. 2015 [35]	Netherlands	Breast	IG: 76 CG: 77	IG: 49.9 (8.4) CG: 51.6 (8.8)	W	CI: Combined CG: Passive Usual Care (no exercise/maintain habitual activity/no specific advice)	20 weeks 2 days/week	EORTC QLQ-C30	IG Pre: 89.4 ± 10.2 Post: 80.3 ± 14.1 CG Pre:84.8 ± 13.8 Post: 68.1 ± 17.6
Mijwel et al. 2018 [73]	Sweden	Breast	GRT/HIIT: 74 GAT/HIIT: 72 CG: 60	RT/HIIT: 52.7 (10.3) AT/HIIT: 54.4 (10.3) CG: 52.6 (10.2)	W	GRT/HIIT + RT GAT/HIIT + AT CG: Usual Care with General Recommendations (ACSM, WHO, healthy lifestyle advice)	16 weeks 2 days/week	EORTC QLQ-C30	GRT/HIIT Pre: 63.56 ± 24.97 Post: 63.68 ± 19.11 GAT/HIIT Pre: 66.67 ± 20.90 Post: 63.48 ± 19.06 CG Pre: 68.84 ± 21.66 Post: 59.91 ± 19.03
Ammitzbøllet al. 2019 [38]	Denmark	Breast	IG: 82 CG: 76	IG: 53 (33–73) CG: 52 (30–74)	W	IG: RT CG: Passive Usual Care (no exercise/maintain habitual activity/no specific advice)	20 weeks 2 days/week	EORTC QLQ-C30	IG Δ change IC95% 1.5 (5.5; 8.5) CG Δ changeIC95% 0.00 Reference
Baglia et al. 2019 [64]	USA	Breast	IG: 61 CG: 60	IG: 61.2 (7.09 CG: 62.0 (7.0)	W	IG: RT + AT CG: Passive Usual Care (no exercise/maintain habitual activity/no specific advice)	52 weeks 2 days/week	FACT-B	IG Pre: 102.2 ± 18.07 Δ change IC95% + 10.2 (6.7 to13.6) CG Pre: 100.2 ± 18.7 Δ change IC95% + 2.0 (1.8 to 5.7)
Mijwel et al. 2019 [36]	Sweden	Breast	GRT/HIIT: 74 GAT/HIIT: 72 CG: 60	GRT/HIIT: 52.7 (10.3) GAT/HIIT: 54.4 (10.3) CG: 52.6 (10.2)	W	RT: HIIT + RT AT HIIT: HIIT + AT CG: Usual Care with General Recommendations (ACSM, WHO, healthy lifestyle advice)	16 weeks 2 days/week	EORTC QLQ-C30	GRT/HIIT Pre: 63.56 ± 24.97 Post: 63.85 ± 19.88 GAT/HIIT Pre: 66.67 ± 20.90 Post: 63.75 ± 20.29 CG Pre: 68.84 ± 21.66 Post: 59.52 ± 19.62
Ndjavera et al. 2020 [37]	UK	Prostate	IG: 24 CG: 26 ITT	IG: 71.4 (5.4) CG: 72.5 (4.2)	M	IG: Combined CG: Passive Usual Care (no exercise/maintain habitual activity/no specific advice)	12 weeks 2 days/week	FACT-P	IG Pre: 119.0 ± 19.0 Post: 123.0 ± 22.0 CG Pre: 123.0 ± 16.0 Post: 123.0 ± 19.0
Pereira-Rodríguez et al. 2020 [77]	Mexico	Breast	GMICT: 80 GHIIT: 70 CG: 66	MICT: 51 (4) HIIT: 55 (5) CG: 53 (7)	W	MICT: Combined HIIT: HIIT + RT CG: Usual Care with General Recommendations (ACSM, WHO, healthy lifestyle advice)	36 weeks 3 days/week	EORTC QLQ-C30	GMICT Pre: 60.7 ± 16.7 Post: 68.7 ± 13.6 GHIIT Pre: 58.6 ± 17.0 Post: 125 ± 11.8 CG Pre: 64.5 ± 12.96 Post: 63.3 ± 13.4
Piraux et al. 2020 [78]	Belgium	Prostate	GHIIT: 24 GRES: 24 CG: 24	HIIT: 67.4 (8.9) RES: 67.9 (7.1) CG: 71.9 (8.1)	M	HIIT: HIIT RT: RT CG: Usual Care with General Recommendations (ACSM, WHO, healthy lifestyle advice)	5–8 weeks 3 days/week	FACT-G	GHIIT Pre 86.0 [78.2; 91.0] Post: 89.0 [78.5; 92.5] GRES Pre: 83.5 [75.5; 91.8] Post: 82.5 [72.3; 93.9] CG Pre: 79.3 [73.3; 83,3] Post: 77.9 [67.5; 85.4]
Scott et al. 2020 [80]	USA	Breast	GLET: 58 GNLET:59 CG:57	LET: 58 (9) NLET: 59 (9) CG: 58 (9)	W	LET: AT NLET: AT + HIIT CG: active structured controls (Stretching)	16 weeks 3–4 days/week	FACT-B	GLET Pre: 104.8 ± 17.2 Post: 107.8 ± 20.7 GNLET Pre: 111.6 ± 14.1 Post: 116.7 ± 14.0 CG Pre: 107.6 ± 16.3 Post: 108.9 ± 16.2
Gal et al. 2021 [66]	Netherlands	Breast	IG: 68 CG: 114	IG: 58.0 (9.8) CG: 58.3 (9.5)	W	IG: Combined CG: Passive Usual Care (no exercise/maintain habitual activity/no specific advice)	12 weeks 2 days/week	EORTC QLQ-C30	IG Δ change IC95% 0.8 (−1.4 to 3.0) CG Δ change IC95% 0.00 Reference
Moraes et al. 2021 [75]	Brazil	Breast	IG: 13 CG: 13	IG: 55.0 (5.8) CG: 54.3 (5.2)	W	IG: RT CG: Passive Usual Care (no exercise/maintain habitual activity/no specific advice)	8 weeks 1 day/week	EORTC QLQ-C30	IG Pre: 54.6 ± 27.2 Post: 76.81 ± 22.5 CG Pre: 58.5 ± 28.9 Post: 59.85 ± 30.0
Harrison et al. 2022 [68]	USA	Prostate	IG: 13 CG: 13	IG: 65.7 (8.1) CG: 64.4 (8.3)	M	IG: Combined CG: Passive Usual Care (no exercise/maintain habitual activity/no specific advice)	16 weeks 3 days/week	FACT-P	IG vs. CC Δ change IC95% +5.7 [−4.0 to 15.5]
Kang et al. 2022 [70]	Canada	Prostate	IG: 26 CG: 26	63.4 (7.1)	M	IG: HIIT CG: Passive Usual Care (no exercise/maintain habitual activity/no specific advice)	12 weeks 3 days/week	EORTC QLQ-C30	IG Pre: 76.7 ± 13.4 Post: 80.7 ± 13.5 CG Pre: 74.7 ± 14.9 Post: 74.3 ± 16.7
Koevoets et al. 2022 [72]	Netherlands	Breast	IG: 91 CG: 90	IG: 52.1 (8.6) CG: 52.5 (8.7)	W	IG: Combined CG: Passive Usual Care (no exercise/maintain habitual activity/no specific advice)	6 months 4 days/week	EORTC QLQ-C30	IG vs. CC Δ change IC95% 3.96 [1.21 to 6.71]
Antunes et al. 2024 [30]	Portugal	Breast	IG: 47 CG: 46	IG: 49.66 (9.43) CG: 51.02 (9.54)	W	IG: Combined. CG: Passive Usual Care (no exercise/maintain habitual activity/no specific advice)	20–24 weeks 3 days/week	EORTC QLQ-C30 EORTC QLQ-BR23	IG Pre: 85.66 ± 9.15 Post: 82.78 ± 10.65 CG Pre: 87.64 ± 8.52 Post: 76.96 ± 13.98
Cešeiko et al. 2019 [65]	Latvia	Breast	IG: 27 CG: 28	IG: 48.2 (6.7) CG: 49.0 (8.0)	W	IG: RT CG: Usual Care with weekly phone call/maintain activity	12 weeks 2 days/week	EORTC QLQ-C30 EORTC QLQ-BR23	IG Pre: 67.2 ± 15.6 Post: 76.2 ± 14.3 CG Pre: 66.3 ± 16.5 Post: 63.5 ± 14.7
García-Soidán et al. 2015 [67]	Spain	Breast	RTG: 79 AG: 79 ATG: 79 CG: 79	RTG: 63 (7) AG: 62 (6.8) ATG: 64 (7.1) CG: 63 (4.6)	W	IG: RT IAG: Aquagym IG: AT CG: Passive Usual Care (no exercise/maintain habitual activity/no specific advice)	2 years (45 weeks/year) 2 days/week	SF-12	GRT Pre: 45.6 ± 4.2 Post: 47.5 ± 7.8 GIAG Pre: 45.1 ± 4.1 Post: 47.8 ± 7.0 GAT Pre: 44.8 ± 3.8 Post: 47.3 ± 8.5 CG Pre: 43.8 ± 4.5 Post: 46.9 ± 7.4
Klavina et al. 2024 [71]	Latvia	Breast	IG: 17 CG: 20	IG: 48.56 (7.84) CG: 48.53 (8.21)	W	IG: HIIT CG: Passive Usual Care (no exercise/maintain habitual activity/no specific advice)	6 months 2–3 days/week	EORTC QLQ-C30 EORTC QLQ-BR23	GHIIT Pre: 69.6 ± 16.9 Post: 64.67 ± 22.2 CG Pre: 66.9 ± 24.58 Post: 68.51 ± 20.67
Monazzami et al. 2020 [74]	Iran	Breast	IG: 22 CG: 20	IG: >30 CG: >30	W	IG: Combined CG: Passive Usual Care (no exercise/maintain habitual activity/no specific advice)	8 weeks 3 days/week	MQOL	IG Pre: 4.52 ± 0.89 Post: 7.52 ± 0.67 CG Pre: 4.94 ± 0.53 Post: 5.23 ± 0.55
Nilsen et al. 2015 [39]	Norway	Prostate	IG: 30 CG: 28	IG: 66 (6.6) CG: 66 (5)	M	IG: RT CG: Passive Usual Care (no exercise/maintain habitual activity/no specific advice)	16 weeks 3 days/week	EORTC QLQ-C30	IG Pre: 76.5 ± 17.3 Post: 79.6 ± 17.0 CG Pre: 66.7 ± 19.6 Post: 78.9 ± 20.7
Paulo et al. 2019 [76]	Brazil	Breast	IG: 18 CG: 18	IG: 63.2 (7.1) CG: 66.6 (9.6)	W	IG: Combined CG: active structured controls (Stretching)	9 months 3 days/week a	EORTC QLQ-C30 EORTC QLQ-BR23	IG Pre: 70.6 ± 14.1 Post: 84.4 ± 9.8 CG Pre: 66.6 ± 15.2 Post: 73.8 ± 11.1
Shobeiri et al. 2016 [41]	Iran	Breast	IG: 27 CG: 26	IG: 42.70 (9.60) CG: 43.50 (8.60)	W	IG: AT CG: Passive Usual Care (no exercise/maintain habitual activity/no specific advice)	10 weeks 2 days/week	EORTC QLQ-C30 EORTC QLQ-BR23	IG Pre: 48.76 ± 24.96 Post: 81.79 ± 16.34 CG Pre: 47.75 ± 15.73 Post: 52.88 ± 14.51
Hojan & Milecki 2020 [69]	Poland	Prostate	IG: 36 CG: 36	IG: 65.7 (6.2) CG: 67.9 (4.9)	M	IG: Combined CG: Passive Usual Care (no exercise/maintain habitual activity/no specific advice)	8 weeks 5 days/week	FACT-G	IG Pre: 70.7 ± 2.1 Post: 73.3 ± 6.3 CG Pre: 70.2 ± 1.9 Post: 55.3 ± 3.9
IG: intervention group. CG: control group. EORTC QLQ-C30 = European Organisation for Research and Treatment of Cancer Quality-of-Life Questionary Core 30. SF-36 = Short Form 36. FACT-B = Functional Assessment of Cancer Therapy—Breast. FACT-P = Functional Assessment of Cancer Therapy—Prostate. FACT-G = Functional Assessment of Cancer Therapy—General. EORTC QLQ-BR23 = European Organisation for Research and Treatment of Cancer Quality of Life Questionnaire Breast Cancer. SF-12 = Short Form-12. MQOL = McGill Quality of Life Questionnaire. RT = resistance training. AT = aerobic training. HIIT = High-intensity Interval training. MICT = Moderate-Intensity Continuous Training. ET = Endurance Training. LET = Linear Exercise Therapy. NLET = Non-Linear Exer.									

## Appendix C. Timing of Exercise Interventions in Breast and Prostate Cancer Studies

**Study**	**Cancer Type**	**Intervention Timing**	**During Tx**	**Post-Tx < 6 m**	**Post-Tx 6–12 m**	**Post-Tx 12–24 m**	**Post-Tx** **>** **24 m**
Schmidt et al. 2015 [33]	Breast	During chemotherapy	✔				
Schmidt et al. 2015 [79]	Breast	During chemotherapy	✔				
Travier et al. 2015 [34]	Breast	During chemotherapy	✔				
Van Waart et al. 2015 [35]	Breast	During chemotherapy	✔				
Mijwel et al. 2018 [73]	Breast	During chemotherapy	✔				
Ammitzbøllet al. 2019 [38]	Breast	Post cx		✔			
Baglia et al. 2019 [64]	Breast	Post-cx During hormonal treatment				✔	
Mijwel et al. 2019 [36]	Breast	Post cx During chemotherapy	✔				
Hojan &Milecki 2020 [69]	Prostate	During radiotherapy and ADT treatment	✔				
Ndjavera et al. 2020 [37]	Prostate	During ADT treatment	✔				
Pereira-Rodríguez et al. 2020 [77]	Breast	During or after oncologic treatment (timing not specified; treatment not interrupted)	✔				
Piraux et al. 2020 [78]	Prostate	During radiotherapy	✔				
Scott et al. 2020 [80]	Breast	Post-treatment					✔
Gal et al. 2021 [66]	Breast	Post-treatment Possibly concomitant with hormonal therapy		✔			
Moraes et al. 2021 [75]	Breast	Post-treatment Possibly concomitant with hormonal therapy		✔			
Harrison et al. 2022 [68]	Prostate	4 weeks prior to ADT + enzalutamide, continuing during treatment	✔				
Kang et al. 2022 [70]	Prostate	Active surveillance	✔				
Antunes et al. 2024 [30]	Breast	During chemotherapy	✔				
Cešeiko et al. 2019 [65]	Breast	During chemotherapy	✔				
García-Soidán et al. 2015 [67]	Breast	Post-treatment		✔			
Klavina et al. 2024 [71]	Breast	During chemotherapy	✔				
Monazzami et al. 2020 [74]	Breast	During hormonal treatment	✔				
Nilsen et al. 2015 [39]	Prostate	During ADT treatment	✔				
Paulo et al. 2019 [76]	Breast	During hormonal treatment	✔				
Shobeiri et al. 2016 [41]	Breast	Post non-hormonal treatment; possibly concomitant with hormonal therapy		✔			
Note: The “✔” symbol indicates compliance with the corresponding feature.							
